# Barrier to Entry: How Health Canada’s Medical Device Single Audit Program Limits Patient Access to New Technology in Canada

**DOI:** 10.1177/19160216261472799

**Published:** 2026-09-01

**Authors:** Andrew Kokavec, Tina Meisami, Brian W. Rotenberg

**Affiliations:** 1 Department of Otolaryngology—Head and Neck Surgery, Schulich School of Medicine and Dentistry, Western University, London, ON, Canada; 2 Surgical and Dental Sleep Medicine, University Health Network, Toronto, ON, Canada

**Keywords:** adult rhinology, clinical, allergy/rhinology, cost analysis, clinical, allergy/rhinology, outcomes/cost-effectiveness, clinical, allergy/rhinology, sleep apnea, clinical, allergy/rhinology, evidence-based medicine, clinical research, health policy, clinical research, sleep medicine, CPAP therapy, sleep medicine, obstructive sleep apnea, sleep medicine, surgical treatment of obstructive sleep apnea, sleep medicine

## Abstract

In this article, we look at the approval process for medical devices in Canada, and how the Medical Device Single Audit Program (MDSAP) has made it unfeasible for small manufactures to enter the Canadian market. Specifically, we explore how MDSAP has been a barrier in Canada for the entry of hypoglossal nerve stimulators (HGNS), a proven technology with a strong track record in other markets for the treatment of obstructive sleep apnea. This is ultimately preventing Canadian patients access to life-saving medical devices. We hope this commentary will shed light on this barrier and prompt Health Canada to explore other avenues for the approval of medical devices as is the case in other developed countries.

## Key Message

- Hypoglossal nerve stimulation is a proven therapy for obstructive sleep apnea, but it remains inaccessible to Canadians.
- Health Canada’s rigid regulatory framework—not clinical safety—is the barrier to entry. By mandating the costly and complex Medical Device Single Audit Program as the exclusive pathway for approval, Canada’s “one-size-fits-all” approach economically deters smaller, specialized innovators from entering a relatively small market.
- Canada must adopt flexible regulatory pathways to restore patient access to medical innovation. To fix this policy, Health Canada should implement a mutual recognition or reliance framework that accepts existing approvals from peer regulatory bodies (such as the FDA, EMA, or TGA), aligning Canada with international norms and improving access to life-saving technology.

## The Unmet Need in Sleep Apnea: Beyond CPAP Adherence

Obstructive sleep apnea (OSA) is a pervasive chronic condition in Canada, with recent data suggesting 28.1% of the population aged 45 to 85 meets the diagnostic criteria.^1^ Beyond the immediate impact on sleep quality, untreated OSA is a driver of systemic burden, correlating with higher incidences of stroke, diabetes, healthcare resource utilization, and motor vehicle accidents. While Continuous Positive Airway Pressure (CPAP) is often prescribed as first-line treatment, its real-world utility is limited by poor long-term adherence.^2,3^

For patients who are intolerant to CPAP, the hypoglossal nerve stimulation (HGNS) procedure has emerged globally as an effective alternative. HGNS is an implantable device, similar to a pacemaker, which prevents airway collapse through synchronized stimulation of the hypoglossal nerve, in essence becoming a surgical CPAP. HGNS is indicated for patients with moderate-to-severe OSA, who are CPAP-intolerant or non-adherent and meet anatomical criteria.^4^ High-quality evidence, including a 2024 systematic review, demonstrates that HGNS significantly reduces the Apnea-Hypopnea Index (AHI), is cost-effective, and improves quality of life, with adherence rates far exceeding those of CPAP.^5,6^ Yet, despite its mainstream adoption in the United States, Europe, and Japan, this life-saving technology remains inaccessible to Canadians. The barrier is not clinical safety or efficacy, but rather an overly rigid regulatory framework: Health Canada’s Medical Device Single Audit Program (MDSAP).

## The Medical Device Single Audit Program (MDSAP)

To understand the unavailability of HGNS in Canada, one must examine the evolution of medical device regulation. Developed under the International Medical Device Regulators Forum (IMDRF) starting in 2012, MDSAP was designed to streamline global regulation. It allows a single audit by a recognized third-party organization to satisfy regulatory requirements of the 5 participating jurisdictions: Australia, Brazil, Canada, Japan, and the United States.^7^

On January 1, 2019, Health Canada mandated a transition from the Canadian Medical Devices Conformity Assessment System (CMDCAS) to MDSAP. For all manufacturers of Class II, III, and IV devices, MDSAP became the sole pathway for Canadian market entry.^7^ Devices are classified based on increasing risk to patient health with implantable devices falling into Class IV.

The policy intent was clear: to promote international harmonization, improve regulatory oversight, and reduce the burden of multiple redundant audits. However, the practical application has introduced significant friction for smaller manufacturers. Reports indicate that MDSAP audit fees are often 2 to 3 times higher than those under the previous CMDCAS system, with annual certification costs frequently ranging between $35 000 and $80 000 USD.^8^ Furthermore, for companies attempting to enter a single market, the administrative burden of satisfying the distinct regulatory nuances of 5 different countries in a single audit adds layers of complexity and preparation time.

## Harmonization at the Cost of Flexibility

The MDSAP model is not inherently flawed; rather, it is differentially impactful based on the scale of the manufacturer. For large, multinational corporations—such as Medtronic which produces high-volume cardiac implants that were Health Canada approved—MDSAP makes economic and logistical sense. With annual revenues in the billions of dollars, these entities have the capital to absorb high audit fees and the infrastructure to manage complex compliance requirements. For them, a single audit that unlocks 5 major markets is an efficiency gain and regulatory fees become a small issue to manage.

However, this “one-size-fits-all” mandate creates a barrier for smaller, specialized companies. For an innovator with a niche technology, such as those producing the HGNS products, the Canadian market is often a small fraction of the global one. When the cost and complexity of the mandatory Canadian MDSAP process outweighs the potential revenue from the Canadian market, the rational economic decision is to bypass Canada entirely, and that is exactly what has happened.

## Global Alignment: Lessons from Peer Regulators

The unavailability of HGNS in Canada to treat sleep apnea is a direct manifestation of this policy failure. Unlike the United States, Japan, Australia, and Brazil—where MDSAP is voluntary and alternative pathways exist—Canada stands alone in making MDSAP the exclusive mechanism for approval. For example, while Australia’s Therapeutic Goods Administration (TGA) encourages the use of MDSAP, it also allows manufacturers to leverage existing approvals in similar markets as part of their comparable overseas regulator pathway.^9^ This streamlines approval for devices that are already proven therapies in other markets. As another example, the larger and more lucrative EU market is not a full member of MDSAP, and manufacturers must undergo an EU MDR-CE audit: to subsequently enter the Canadian market, the same companies must pay for MDSAP on top of their EU cost, whereas in other regions, they can “piggyback” off their EU certification (eg, Australia often accepts EU CE marking as proof of safety, avoiding a full duplicate audit). That work-around is not allowed by Health Canada.^9^

Although Health Canada’s Special Access Program (SAP) provides a theoretical, case-by-case pathway for unlicensed devices like HGNS when all conventional treatments have failed,^10^ its highly restrictive criteria render it an inadequate substitute for systemic regulatory approval. This exception-based model shifts administrative burden onto individual healthcare providers—who must submit extensive clinical justifications and reporting obligations for each patient—all without any guarantee of manufacturer supply or provincial funding.

## Conclusion

Manufacturers of HGNS devices have explicitly indicated to the authors that the decision to forgo Health Canada approval is driven by the unfeasibility of the MDSAP process relative to the Canadian market size. Specialized manufacturers face a difficult choice: invest high costs and significant administrative hours for a mandatory audit to enter the relatively small Canadian market or simply focus on the larger US and European markets where regulatory pathways are more flexible or already cleared. Consequently, Canadian patients are denied access to proven therapeutic modalities not because a device is unsafe or uncertain, but because the administrative pathway through Health Canada is economically prohibitive for the manufacturer.

The transition to MDSAP has achieved its goal of harmonization for large industry players, but it has also created a roadblock against specialized innovation. By mandating MDSAP as the sole pathway for approval, Health Canada has created a prohibitive regulatory process for smaller vendors that doesn’t exist in other countries. We call on Health Canada to reconsider this exclusionary stance. Reintroducing alternative regulatory pathways for smaller manufacturers—such as a mutual recognition or reliance framework for devices already approved by the FDA, EMA, or TGA—would align Canada with its international peers and, more importantly, allow patient access to life-saving technologies such as hypoglossal nerve stimulation to treat sleep apnea.
